# Adverse effects on growth performance and bone development in nursery pigs fed diets marginally deficient in phosphorus with increasing calcium to available phosphorus ratios

**DOI:** 10.1093/jas/skaa325

**Published:** 2020-10-05

**Authors:** Spenser L Becker, Stacie A Gould, Amy L Petry, Leah M Kellesvig, John F Patience

**Affiliations:** 1 Department of Animal Science, Iowa State University, Ames, IA; 2 Iowa Pork Industry Center, Iowa State University, Ames, Iowa; 3 Vita Plus Corporation, Madison, WI

**Keywords:** bone mineral content, dual x-ray absorptiometry, swine

## Abstract

The objective of this experiment was to evaluate the growth performance and bone mineral content (BMC) of nursery pigs in response to increasing total calcium (Ca) to available phosphorus (**aP**) ratios in diets containing phytase (250 FTU/kg; Natuphos E, BASF, Florham Park, NJ). A total of 480 nursery pigs (body weight (**BW**) = 5.7 ± 0.6 kg) with 10 pigs per pen and 7 pens per treatment (6 pens fed 2.75:1 diet) were allotted to seven treatments consisting of increasing ratios of calcium to available phosphorus (**Ca:aP**): 1.25, 1.50, 1.75, 2.00, 2.25, 2.50, and 2.75. From day −7 to 0, pigs were fed a common diet. They were then fed the treatment diets during two experimental phases from day 1 to 14 and 15 to 28, respectively. Available P was formulated to 0.33% and 0.27% (approximately 90% of requirement) in dietary phases 1 and 2, respectively. BW, average daily gain (**ADG**), average daily feed intake (**ADFI**), and gain-to-feed ratio (**G:F**) were determined. BMC of the femur was measured on day 28 on one pig per pen using dual x-ray absorptiometry. Data were analyzed as a linear mixed model using PROC MIXED (SAS, 9.3). Orthogonal polynomial contrasts were used to determine the linear and quadratic effects of increasing the Ca:aP. Over the 28-d experimental period, increasing Ca:aP resulted in a linear decrease in ADG (353, 338, 328, 304, 317, 291, and 280 g/d; *P* < 0.01), ADFI (539, 528, 528, 500, 533, 512, and 489 g/d; *P* < 0.05), and G:F (0.68, 0.66, 0.64, 0.62, 0.61, 0.59, and 0.58; *P* < 0.01). Increasing Ca:aP also resulted in decreased BW on days 14 and 28 (*P* < 0.01). The BMC of the femur decreased with increasing Ca:aP (6.2, 6.3, 5.7, 5.9, 5.5, 5.6, and 5.3 g; *P* < 0.05). Regression analysis explained the impact of Ca:aP as follows on ADG (ADG [g/d] = 339 − 36x; *r*^2^ = 0.81), G:F (G:F = 0.61 – 0.03x; *r*^2^ = 0.72), and BMC (BMC [g] = 6.4 – 0.27x; *r*^2^ = 0.43), where x is the Ca:aP. In conclusion, all outcomes indicated that any level of calcium above the minimum used in this experiment impaired growth performance and skeletal development. Further research using even lower levels of dietary Ca is warranted.

## Introduction

Calcium (Ca) and phosphorus (P) are the two most abundant minerals in the body of the pig and are required for many important physiological functions (Oster et al., 2016). The vast majority of Ca—about 99%—is not only present in skeletal tissues (Nielson, 1972), but it also fulfills other very important roles involving blood clotting, nerve impulse transmission, muscle contractility, and fluid balance, to name a few (Pravina et al., 2013). In contrast, only about 77.5% of the P in the body can be found in skeletal tissues (Nielson, 1972); it is also involved in a wide array of metabolic functions, including energy metabolism, protein synthesis, signal transduction, acid–base homeostasis, and cell membrane polarity (Oster et al., 2016). The fact that the P status of the pig may influence adaptive immune function is further evidence of the central importance of maintaining an adequate dietary supply of this critical mineral element (Heyer et al., 2015).

The ratio of calcium to phosphorus (**Ca:P**) in bone is about 2.1:1; this is tightly regulated by the finite chemical structure of hydroxyapatite which, along with collagen, constitutes most of the structure of bone (Cromwell, 2005). Maintenance of desirable blood levels of Ca and P is the consequence of the intricate balance between renal excretion, enteral absorption, and osseous mobilization or accumulation (Berndt and Kumar, 2009). These are all under some degree of control by the endocrine system, including calcium-binding protein, parathyroid hormone, vitamin D, and calcitonin.

If either Ca or P is present in the diet in excess or deficit relative to requirement, compromised utilization of the other may occur (Létourneau-Montminy et al., 2015). Specifically, overfeeding Ca impairs P absorption, at least in part due to the formation of insoluble tricalcium phosphate, leading to negative impacts on skeletal development and growth performance (Cromwell, 2005). Notably, the Ca:P in the diet is increasingly important when P is close to or below requirement (Hays, 1976; Peo, 1976; Létourneau-Montminy et al., 2015; Wu et al., 2018). This is an important concept because most practical diets are formulated to be as close as possible to requirement, in order to minimize the quantity of P excreted in the manure (Bridges et al., 1995), to preserve finite global P reserves (Edixhoven et al., 2013), and to minimize diet cost; the latter is important as P is the third most expensive nutrient in the diet after carbohydrates (energy) and protein (Patience, 2017). It, therefore, begs the question as to how severely performance and skeletal development are impaired if P inadvertently falls slightly below requirement and, particularly, when Ca is present in relative excess.

There is a lack of clarity on the dose–response to a wide calcium:available phosphorus ratio (**Ca:aP**) when available phosphorus (**aP**) is marginally deficient. The importance of the ratio is well known, but the quantitative relationship between Ca:aP and growth performance and bone development is much less clear (Wu et al., 2018). Therefore, the objective of this experiment was to characterize the nature of this relationship by titrating an increasingly wide Ca:aP against pig performance and bone development when the basal diet was marginally deficient in aP. This objective tested the hypothesis that even a narrow Ca:aP will negatively impact growth performance and bone development, and as this ratio widens, the effect would be amplified at an increasing rate. 

## Materials and Methods

All experimental procedures adhered to the principles for the ethical and humane use of animals according to the *Guide for the Care and Use of Agricultural Animals in Research and Teaching* (FASS, 2010) and were approved by the Iowa State University Animal Care and Use Committee (IACUC #18-99).

### Animals, housing, and experimental design

A total of 480 weanling pigs (5.7 ± 0.6 kg body weight [**BW**]; L337 × Camborough, PIC, Inc., Hendersonville, TN) were purchased and transported to the Iowa State University Swine Nutrition Farm (Ames, IA). Upon arrival, pigs were individually weighed, ear-tagged, and vaccinated for K88^+^  *Escherichia coli* via a water-delivered vaccine (Arko Laboratories, Jewell, IA). Pigs were blocked by initial weight into seven blocks, and pens were randomly assigned to one of seven dietary treatments. Pigs were housed 10 pigs per pen and 6 or 7 pens per treatment; with 48 pens available and seven treatments, one treatment was randomly chosen to have 1 less block(pen) than the others. Pens contained mixed sexes with the same number of barrows and gilts per pen across all treatments within each block.

### Diets and feeding

Pens (1.2 × 2.4 m) were equipped with a four-space dry self-feeder and two nipple waterers to provide ad libitum access to feed and water. Pigs received a common standard nursery diet from day −7 to 0 during the pretest acclimation period. They then received the experimental diets over two phases from day 1 to 14 (phase 1) and day 14 to 28 (phase 2), respectively. Diets were formulated to meet or exceed the NRC (2012) nutrient recommendations, with the exception of Ca and P. Diets consisted of increasing Ca:aP: 1.25, 1.50, 1.75, 2.00, 2.25, 2.50, and 2.75. Available P was kept constant in each diet and formulated to be 0.33% and 0.27% (about 90% of requirement; NRC, 2012) in dietary phases 1 and 2, respectively.

### Data and sample collection

Pig BW and feed intake were measured on days 0 and 28 of the experiment to calculate average daily gain (**ADG**), average daily feed intake (**ADFI**), and gain-to-feed ratio (**G:F**). On day 28, one gilt from each pen representing the average pen weight was euthanized via captive bolt stunning followed by exsanguination. The right femur was harvested, carefully cleaned, and stored at −20 °C for later analysis. Femurs were analyzed for bone mineral content (**BMC**) using dual-energy X-ray absorptiometry (Hologic Discovery A, Bedford, MA, USA).

Multiple diet subsamples were collected as each feed batch was unloaded from the mixer. Samples were carefully and thoroughly homogenized, subsampled, and stored at −20 °C until later analysis.

### Analytical methods

All ingredients containing Ca and P were sampled and analyzed for Ca and P content prior to the formulation of the experimental diets (Eurofins Scientific, Des Moines, IA; AOAC 984.27, 927.02, 985.01, and 965.17 modified). The results of these assays were then utilized in the formulation of the diets (BESTMIX Feed Formulation, Adifo Software, Maldegem, Belgium). Complete feed samples were analyzed for total Ca and P (Tables 1 and 2; Eurofins Scientific, Des Moines, IA) using the same procedures as previously described.

**Table 1.** Ingredient and nutrient composition of the experimental diets: phase 1 (as-fed basis, %)^1^

	Ca:aP						
Item	1.25	1.50	1.75	2.00	2.25	2.50	2.75
Ingredient, %							
Corn	46.85	46.64	46.44	46.23	46.02	45.82	45.61
Soybean meal, 46.5% crude protein	22.50	22.50	22.50	22.50	22.50	22.50	22.50
Oats, steam rolled	10.00	10.00	10.00	10.00	10.00	10.00	10.00
Whey permeate	10.00	10.00	10.00	10.00	10.00	10.00	10.00
Enzyme-treated soybean meal	6.00	6.00	6.00	6.00	6.00	6.00	6.00
Soybean oil	2.00	2.00	2.00	2.00	2.00	2.00	2.00
l-Lysine HCl	0.59	0.59	0.59	0.59	0.59	0.59	0.59
dl-Methionine	0.28	0.28	0.28	0.28	0.28	0.28	0.28
l-Threonine	0.25	0.25	0.25	0.25	0.25	0.25	0.25
l-Tryptophan	0.03	0.03	0.03	0.03	0.03	0.03	0.03
l-Valine	0.09	0.09	0.09	0.09	0.09	0.09	0.09
Sodium chloride	0.61	0.61	0.61	0.61	0.61	0.61	0.61
Limestone	0.24	0.45	0.65	0.86	1.07	1.27	1.48
Monocalcium phosphate	0.30	0.30	0.30	0.30	0.31	0.31	0.31
Phytase^2^	0.01	0.01	0.01	0.01	0.01	0.01	0.01
Vitamin mineral premix^3^	0.25	0.25	0.25	0.25	0.25	0.25	0.25
Calculated nutrient levels							
Metabolizable energy, kcal/kg	3,461	3,452	3,446	3,439	3,433	3,426	3,417
Available P, %	0.33	0.33	0.33	0.33	0.33	0.33	0.33
STTD P, %	0.36	0.36	0.36	0.36	0.36	0.36	0.36
Standardized ileal digestible Lys, %	1.35	1.35	1.35	1.35	1.35	1.35	1.35
Standardized ileal digestible Met + Cys, %	0.78	0.78	0.78	0.78	0.78	0.78	0.78
Standardized ileal digestible Thr, %	0.85	0.85	0.85	0.85	0.85	0.85	0.85
Standardized ileal digestible Trp, %	0.24	0.24	0.24	0.24	0.24	0.24	0.24

^1^Phase 1 diet was fed from day 0 to 14 of the experiment.

^2^Natuphos E, BASF, Florham Park, NJ.

^3^Provided a minimum per kg of diet: 2,000 IU of vitamin A; 300 IU of vitamin D3; 25 IU of vitamin E; 0.90 mg of menadione (to provide vitamin K); 3 mg of riboflavin; 10 mg of d-pantothenic acid; 0.01 mg of vitamin B_12_, and 15 mg of niacin, 110 mg of Fe (ferrous sulfate); 2,400 mg of Zn (200 mg/kg as zinc sulfate and 2,200 mg/g as zinc oxide); 50 mg of Mn (manganese sulfate); 20 mg of Cu (copper sulfate); 0.9 mg of I (calcium iodate); and 0.3 mg of Se (sodium selenite).

**Table 2.** Ingredient and nutrient composition of the experimental diets: phase 2 (as-fed basis, %)

Item	1.25	1.50	1.75	2.00	2.25	2.50	2.75
Ingredient, %							
Corn	47.62	47.45	47.28	47.12	46.95	46.78	46.61
Soybean meal, 46.5% crude protein	22.50	22.50	22.50	22.50	22.50	22.50	22.50
Oats, steam rolled	10.00	10.00	10.00	10.00	10.00	10.00	10.00
Whey permeate	10.00	10.00	10.00	10.00	10.00	10.00	10.00
Enzyme-treated soybean meal	6.00	6.00	6.00	6.00	6.00	6.00	6.00
Soybean oil	2.00	2.00	2.00	2.00	2.00	2.00	2.00
l-Lysine HCl	0.44	0.44	0.44	0.44	0.44	0.44	0.44
dl-Methionine	0.21	0.21	0.21	0.21	0.21	0.21	0.21
l-Threonine	0.17	0.17	0.17	0.17	0.17	0.17	0.17
l-Tryptophan	0.01	0.01	0.01	0.01	0.01	0.01	0.01
l-Valine	0.01	0.01	0.01	0.01	0.01	0.01	0.01
Sodium chloride	0.61	0.61	0.61	0.61	0.61	0.61	0.61
Limestone	0.19	0.36	0.52	0.69	0.86	1.03	1.20
Monocalcium phosphate	0.00	0.00	0.00	0.00	0.00	0.00	0.00
Phytase^2^	0.01	0.01	0.01	0.01	0.01	0.01	0.01
Vitamin mineral premix^3^	0.25	0.25	0.25	0.25	0.25	0.25	0.25
Calculated nutrient levels							
Metabolizable energy, kcal/kg	3,468	3,461	3,457	3,450	3,444	3,439	3,433
Available P, %	0.27	0.27	0.27	0.27	0.27	0.27	0.27
STTD P, %	0.31	0.31	0.31	0.31	0.31	0.31	0.31
Standardized ileal digestible Lys, %	1.23	1.23	1.23	1.23	1.23	1.23	1.23
Standardized ileal digestible Met + Cys. %	0.71	0.71	0.71	0.71	0.71	0.71	0.71
Standardized ileal digestible Thr, %	0.77	0.77	0.77	0.77	0.77	0.77	0.77
Standardized ileal digestible Trp, %	0.22	0.22	0.22	0.22	0.22	0.22	0.22

^1^Phase 2 diet was fed from day 14 to 28 of the experiment.

^2^Natuphos E, BASF, Florham Park, NJ.

^3^Provided a minimum per kg of diet: 2,000 IU of vitamin A; 300 IU of vitamin D3; 14 IU of vitamin E; 0.90 mg of menadione (to provide vitamin K); 2.5 mg of riboflavin; 8 mg of d-pantothenic acid; 0.01 mg of vitamin B_12_, and 15 mg of niacin, 110 mg of Fe (ferrous sulfate); 200 mg of Zn (zinc sulfate); 50 mg of Mn (manganese sulfate); 200 mg of Cu (copper sulfate and tri-basic copper chloride); 0.3 mg of I (calcium iodate); and 0.3 mg of Se (sodium selenite).

### Statistical analysis

Data were analyzed according to the following mixed model:

$$\begin{array}{l} Y_{ijk}=\;\mu +\tau_{i}+\upsilon_{j}+e_{ijk} \end{array}$$

where $Y_{ijk}$ is the observed value for kth experimental unit within the ith level of Ca:aP of the jth block for the kth pen; μ is the general mean; $\tau_{i\;}$ is the fixed effect of the ith level of Ca:aP (i = 1 to 7); $\upsilon_{j}$ is the random effect of the jth block (j = 1 to 7); and $e_{ijk}$ is the associated variance as described by the model for $Y_{ijk}$ (l = 1 through 48); assuming $\upsilon_{j}∼N\left(0,\;I\sigma_{\upsilon_{j}}^{2}\right),\;$and $e_{ijkl}∼N(0,\;I\sigma_{e}^{2}$), where *I* is the identity matrix.

Regression parameters for the overall ADG, ADFI, G:F, BMC, and final BW were estimated according to the following model:

$$\begin{array}{l} Y_{ijk}=\beta_{0}+\beta_{1}x_{i}+e_{i} \end{array}$$

where $Y_{ijk}$ is the observed value for kth experimental unit within the ith level of Ca:aP of the jth block for the kth pen; $\beta_{0}$ is the intercept; $\beta_{1}$ is the regression coefficient; $x_{i}$ represents the value of the weighted explanatory continuous variable; and $e_{i}$ is the random error associated with $Y_{ijk}$.

The PROC UNIVARIATE procedure in SAS 9.3 (SAS Inst., Cary, NC) was used to verify the normality and homogeneity of the studentized residuals. The mixed model was analyzed using PROC MIXED, and regression parameters were obtained using PROC GLM. The *r*^2^ for a given model was obtained using leave one out cross (LOOC) validation using PROC GLMSELECT, and the 95% confidence interval bands were applied from the best-fit model. Orthogonal polynomial contrasts were used to determine the linear and quadratic effects of increasing Ca:aP ratio. Least square means were separated using Fisher’s least significant difference test, and treatment differences were considered significant if *P* ≤ 0.05 and trends if 0.05 > *P* ≤ 0.10.

## Results and Discussion

The objective of this experiment was to characterize the nature of the relationship between an increasingly wide Ca:aP and growth performance and BMC when nursery-age pigs are fed diets that are marginally deficient in P. Specifically, the principal investigators wanted to determine how wide the ratio must be before impairment is observed and if a very wide ratio had proportionately more or less impact than a narrower ratio. In other words, is the relationship completely linear or is the expected negative impact intensified at very high ratios? While numerous studies have compared narrow vs. wide Ca:aP when P is deficient (Schlegel and Gutzwiller, 2020), only one other study utilized sufficient dietary treatments to define the slope of the curve (González-Vega et al., 2016b); in this instance, the evaluation involved pigs that were heavier (25 to 50 kg) than those employed in this experiment (6 to 15 kg). In the study reported herein, seven ratios of Ca:aP from 1.25 to 2.75 were evaluated. Available P and total Ca were used due to their common application within the commercial pig industry.

The experiment proceeded without incident; only 1.7% of pigs were removed from the study and none was due to impaired skeletal development or locomotion difficulties. The pretest growth performance of the pigs was within the normal range for this facility and source of pigs and resulted in similar final body weights across treatments on day 0 to the start of the experimental period (Table 3). There were no treatment effects during the pretest period since all of the pigs received a common diet.

**Table 3.** Performance of pigs prior to the start of the experiment^1^

Item	Ca:aP								
	1.25	1.50	1.75	2.00	2.25	2.50	2.75	SEM	Treatment
Initial BW, kg	5.71	5.72	5.72	5.72	5.72	5.73	5.83	0.621	0.999
Final BW, kg	6.24	6.26	6.20	6.21	6.18	6.19	6.30	0.604	0.999
ADG, kg	0.08	0.08	0.07	0.07	0.07	0.07	0.06	0.007	0.666
ADFI, kg	0.11	0.11	0.10	0.11	0.10	0.10	0.10	0.006	0.439
G:F	0.67	0.67	0.66	0.65	0.64	0.64	0.62	0.043	0.977

^1^The pretest period was initiated upon arrival of the pigs to the farm and lasted 7 d, during which a typical phase 1 nursery diet was fed ad libitum. There were 10 pigs per pen, and 6 or 7 pens per treatment, due to the availability of only 48 pens for the experiment. The treatment with six pens per treatment was selected randomly.

The results of the Ca and P assays of the experimental diets confirmed that formulation targets were met across the treatments (Table 4). For example, across all 14 diets, Ca averaged 96.8% of target and P averaged 96.1% of target; both are slightly below formulated levels but well within accepted analytical tolerances (AAFCO, 2000). Achieving assayed levels of Ca and P in the experimental diets that are close to formulated values is critical to experimental precision but can be a particular challenge for Ca and P. For this reason, extra care was given to ingredient pre-assay, thorough mixing of the diets, extensive sampling of each batch of feed, and thorough homogenizing of samples prior to assay, as previously described.

**Table 4.** Formulated and analyzed calcium and phosphorus content of dietary treatments

	Ca:aP						
	1.25	1.50	1.75	2.00	2.25	2.50	2.75
Phase 1^1^							
Formulated, %							
Ca	0.41	0.49	0.58	0.66	0.74	0.83	0.91
P	0.46	0.46	0.46	0.46	0.46	0.46	0.46
Analyzed, %							
Ca	0.41	0.49	0.54	0.66	0.71	0.78	0.85
P	0.45	0.45	0.43	0.46	0.43	0.47	0.43
Phase 2^1^							
Formulated, %							
Ca	0.34	0.41	0.47	0.54	0.61	0.68	0.74
P	0.40	0.40	0.40	0.40	0.40	0.40	0.40
Analyzed, %							
Ca	0.33	0.40	0.45	0.52	0.60	0.64	0.74
P	0.38	0.39	0.37	0.39	0.38	0.36	0.39

^1^Phase 1 was fed from day 0 to 14 and phase 2 was fed from day 14 to 28 of the experiment.

**Figure 1.**
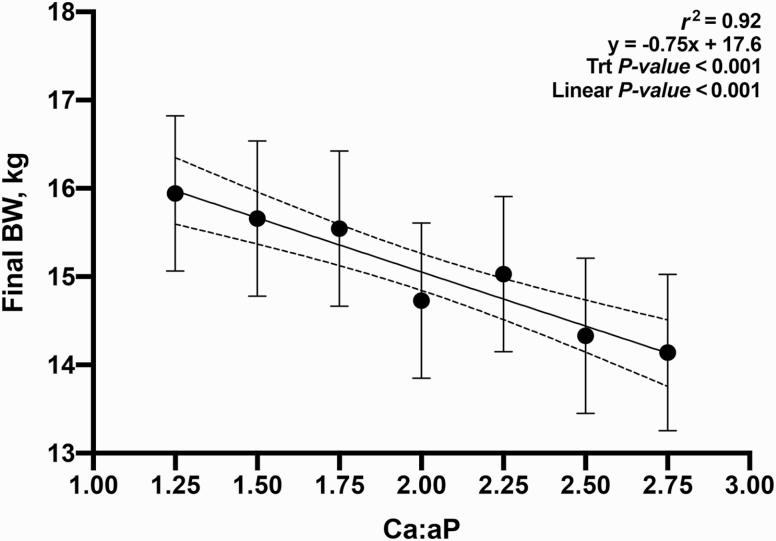
Relationship between the final BW on d 28 and Ca:aP. Data points represent least square means of dietary treatments.

**Figure 2.**
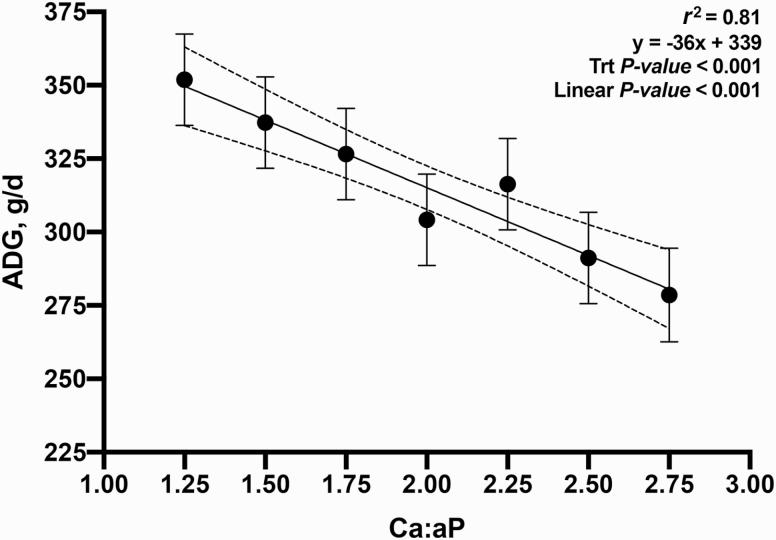
Relationship between day 0 to 28 ADG and Ca:aP. Data points represent least square means of dietary treatments.

**Figure 3.**
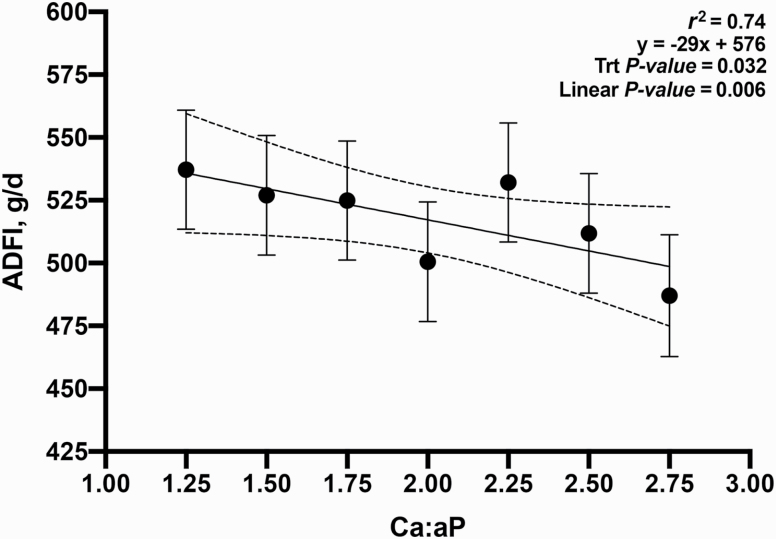
Relationship between day 0 to 28 ADFI and Ca:aP. Data points represent least square means of dietary treatments.

**Figure 4.**
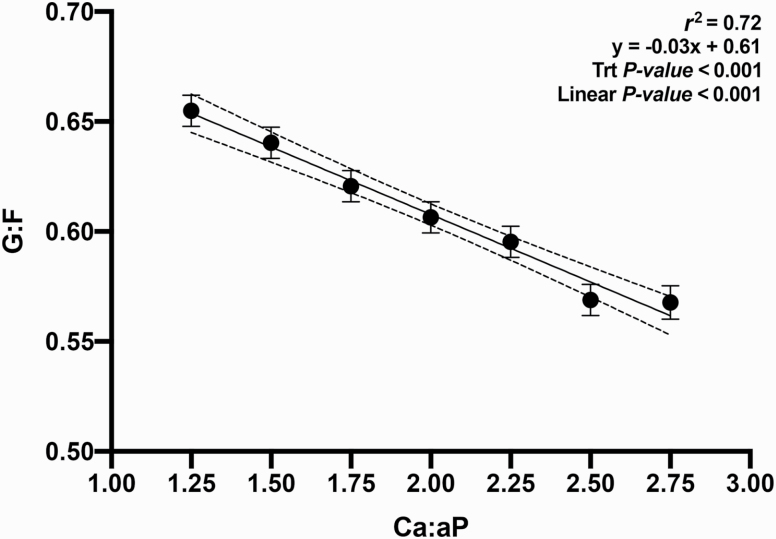
Relationship between day 0 to 28 G:F and Ca:aP. Data points represent least square means of dietary treatments.

**Figure 5.**
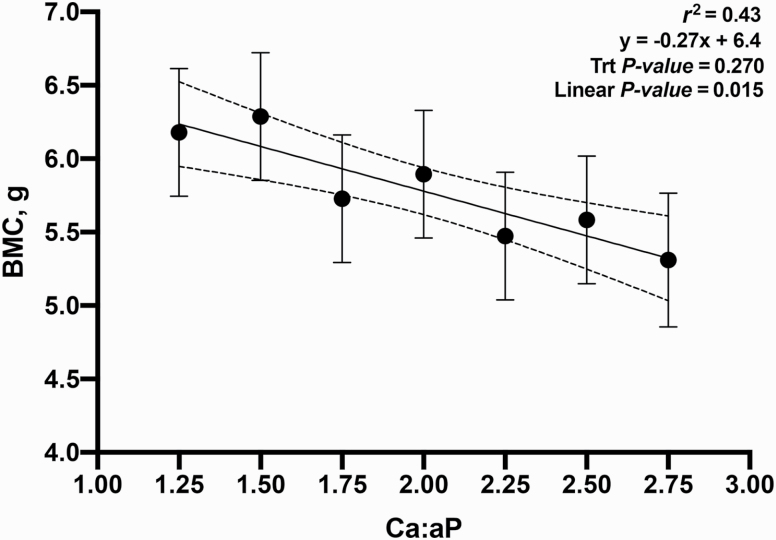
Relationship between BMC and Ca:aP. Data points represent least square means of dietary treatments.

The relationship between Ca:aP was linear for all growth parameters measured: final BW, ADG, ADFI, and G:F (Figures 1–4; *P* < 0.05); in no instance was the response curvilinear or quadratic. These data supported part of our hypothesis; in that as the ratio widened, performance was increasingly impaired. However, it was not supported in that the impact was clearly linear; while growth performance declined as the Ca:aP increased, the slope of the curve did not change. Additionally, the measurement of BMC supported the same conclusion; a small increase in the ratio resulted in impaired bone development and widening this ratio simply extended the range of the response without any change in the slope (Figure 5; *P* < 0.05).

The ideal Ca:aP has not been definitively established for weaned pigs, but great progress has been made in the past half-decade (Gutzwiller et al., 2014; González-Vega et al., 2016b; Merriman et al., 2017; Wu et al., 2018; Lagos et al., 2019b). Research has shown that excess dietary Ca can negatively impact growth performance and bone ash in swine and that this is at least in part dependent on the concentration of dietary P (Qian et al., 1996; Wu et al., 2018; Lagos et al., 2019b). Previous research has demonstrated a detrimental effect of increasing Ca in P-deficient diets on growth performance in nursery (González-Vega et al., 2016a; Lagos et al, 2019a) and grow-finish swine (Liu et al., 1998; Gonzalez-Vega et al., 2016b; Merriman et al., 2017). The reduction in growth performance is possibly explained by the formation of insoluble Ca–P complexes in the gastrointestinal tract when Ca is in excess, resulting in insufficient absorption of P and further exacerbation of the P deficiency (Heaney and Nordin, 2002; Stein et al., 2011). Increasing Ca intake leads to a linear decline in net uptake of P from the gut, irrespective of the level of P in the diet. However, at low dietary P, this decline leads to a negative uptake, which can lead to the adverse effects observed in this study (Heaney and Nordin, 2002). Notably, in pigs and poultry, the negative effects of excess Ca are somewhat mitigated when diets are above the requirement for P (Akter et al., 2018; Wu et al., 2018).

Total Ca to total P ratios (**Ca:tP**) above 1.3:1 in swine diets with low P reduced the growth performance across all stages of production (Reinhart and Mahan, 1986); however, when P was included in the diet above requirement, Ca:tP up to 2.0:1 did not have negative effects on growth performance. When evaluating the Ca:tP in nursery pigs, Qian et al. (1996) reported linear improvements in ADG, ADFI, and feed efficiency when the ratio decreased from 2.0:1 to 1.2:1, regardless of dietary P, which agrees with the results of the current study. Wu et al. (2018) observed no negative impact of Ca:tP from 0.8:1 to 1.6:1 on the growth performance of nursery pigs, but reported reductions in growth performance when this ratio was higher than 1.9:1 and when P was below requirement.

Recent research by Lagos et al. (2019b) demonstrated that if diets are deficient in P (50% of NRC standardized total tract digestible [**STTD**] requirement), then Ca must also be deficient in order to prevent reductions in the growth performance of pigs weighing 11 to 25 kg. Furthermore, Lagos et al. (2019b) determined that if P is included above requirement, then Ca must also be provided above the requirement to improve growth performance, indicating that the effective use of Ca and P is dependent on their ratio as well as their individual concentrations. The authors concluded that the optimal STTD Ca:STTD P ratio to maximize growth performance is 1.40:1 or less for pigs weighing 11 to 25 kg when STTD P is provided at requirement. In this study, reduction of transcellular Ca absorption and increased paracellular Ca absorption were suggested by decreased abundance of genes involved in Ca absorption and tight junction proteins in the small intestine. However, actual protein concentrations were not measured. Nonetheless, the effect of high dietary Ca demonstrates that it may result in impaired intestinal integrity. The authors did not observe any changes in bone ash (g per femur) by increasing dietary Ca if STTD P was deficient, which is in contrast to the current study and surprising. However, the diets utilized by Lagos et al. (2019b) were much more P-deficient than the diets utilized in the present experiment, potentially indicating that P was severely limiting bone deposition. While the Ca:aP cannot be too high, neither can it be too low, as the requirement by the pig for both minerals to support bone growth must be satisfied (Létourneau-Montminy et al., 2012).

Of course, diets are not intentionally formulated or manufactured to be deficient in P, but diet insufficiency can occur under certain circumstances. For example, when pigs experience very low feed intake, daily intake of P can fall below the minimum requirement estimated by the NRC (2012) to be about 2.4 g STTD P per day for pigs of the age employed in this study. Errors in formulation or manufacture, although infrequent, can occur with serious consequences (Crenshaw, 2001). Recently, studies have revealed that the quantity of P released by phytase may have been overestimated, which could also result in P deficiencies (Olsen et al., 2019). Finally, dietary P may be rendered less available in the gastrointestinal tract in the presence of diarrhea (Crenshaw, 2001) or when there is excess Ca in the diet (Heaney and Nordin, 2002).

Our current understanding of P metabolism indicates that pigs do not increase feed intake in response to a primary P deficiency (Misiura et al., 2020). Perhaps, the greater concern is the recent revelation that during circumstances of inadequate P intake, the pig may respond by directing proportionately more of the limited supply of P to protein accretion rather than bone development (Misiura et al., 2020). This may be the result of the pig having some flexibility in bone mass, so that the greater and more urgent need for P would be that used for protein accretion.

Fortunately, a greater understanding of the interaction between Ca and P supply in the diet can be further investigated by measuring urinary and fecal excretion and retention, as well as intestinal and renal transport, such as that demonstrated by Gutierrez et al. (2015). Phosphorus absorption is achieved by both transcellular and paracellular processes; the former dominates when dietary P is below requirement, for example, the conditions of this study (Portale et al., 1989; Saddoris et al., 2010). Enhanced P uptake from the gut when dietary intake is below requirement is a well-known physiological adjustment. When plasma P decreases, Na/P cotransport activity in the gut increases along with activation of 1,25-hydroxylase activity and an elevation in vitamin D_3_ levels (Segawa et al., 2004). In terms of kinetics, the K_m_ for P transport remains unchanged, but V_max_ is elevated. Moreover, renal tissue must also adjust in order to conserve P (Levine et al., 1984; Murer et al., 2003).

In conclusion, the overall growth performance and BMC of nursery pigs are impaired by a Ca:aP at least as low as 1.25:1 when the diet is deficient in P. Indeed, the reduction in growth performance, and in bone development, is progressively worsened as the ratio widens. However, the response of all parameters measured in this study was linear, indicating that while widening the ratio has negative consequences, the impact is not curvilinear. In other words, the severity of the impact did not lessen when the ratio was very wide, but neither did it escalate. Prior to this study, it was known that the Ca:aP was important when dietary P is close to requirement, and that the problem worsens when dietary P is below requirement. The results of this study clearly reveal the linear nature of the relationship between the Ca:aP and pig performance, even when the ratio is quite wide. Bone mineral concentration was similarly affected. These results further confirm the importance of ensuring adequate P and an optimum ratio of Ca:P in swine diets in order to maximize growth performance and skeletal development.

## Abbreviations

ADFI: average daily feed intake
ADG: average daily gain
aP: available phosphorus
BMC: bone mineral content
BW: body weight
Ca:P: calcium to phosphorus ratio
Ca:aP: calcium to available phosphorus ratio
Ca:tP: calcium to total phosphorus ratio
G:F: feed efficiency
STTD: standardized total tract digestible
